# Multiple relaxases contribute to the horizontal transfer of the virulence plasmids from the tumorigenic bacterium *Pseudomonas syringae* pv. savastanoi NCPPB 3335

**DOI:** 10.3389/fmicb.2022.1076710

**Published:** 2022-12-12

**Authors:** Maite Añorga, Miriam Urriza, Cayo Ramos, Jesús Murillo

**Affiliations:** ^1^Institute for Multidisciplinary Research in Applied Biology, Universidad Pública de Navarra (UPNA), Edificio de Agrobiotecnología, Mutilva Baja, Spain; ^2^Área de Genética, Facultad de Ciencias, Universidad de Málaga, Málaga, Spain; ^3^Instituto de Hortofruticultura Subtropical y Mediterránea “La Mayora”, Consejo Superior de Investigaciones Científicas (IHSM-UMA-CSIC), Málaga, Spain

**Keywords:** cytokinin biosynthesis, type III effectors, olive knot disease, type IV secretion system, mating pair formation, *Pseudomonas amygdali*, *Pseudomonas* savastanoi, conjugation (mating)

## Abstract

*Pseudomonas syringae* pv. savastanoi NCPPB 3335 is the causal agent of olive knot disease and contains three virulence plasmids: pPsv48A (pA), 80 kb; pPsv48B (pB), 45 kb, and pPsv48C (pC), 42 kb. Here we show that pB contains a complete MPF_T_ (previously type IVA secretion system) and a functional origin of conjugational transfer adjacent to a relaxase of the MOB_P_ family; pC also contains a functional *oriT*-MOB_P_ array, whereas pA contains an incomplete MPF_I_ (previously type IVB secretion system), but not a recognizable *oriT*. Plasmid transfer occurred on solid and in liquid media, and on leaf surfaces of a non-host plant (*Phaseolus vulgaris*) with high (pB) or moderate frequency (pC); pA was transferred only occasionally after cointegration with pB. We found three plasmid-borne and three chromosomal relaxase genes, although the chromosomal relaxases did not contribute to plasmid dissemination. The MOB_P_ relaxase genes of pB and pC were functionally interchangeable, although with differing efficiencies. We also identified a functional MOB_Q_ mobilization region in pC, which could only mobilize this plasmid. Plasmid pB could be efficiently transferred to strains of six phylogroups of *P. syringae sensu lato*, whereas pC could only be mobilized to two strains of phylogroup 3 (genomospecies 2). In two of the recipient strains, pB was stably maintained after 21 subcultures in liquid medium. The carriage of several relaxases by the native plasmids of *P. syringae* impacts their transfer frequency and, by providing functional diversity and redundancy, adds robustness to the conjugation system.

## Introduction

Approximately three quarters of the protein families in proteobacterial genomes have been acquired by horizontal gene transfer (HGT) during evolution (Popa and Dagan, 2011). In more recent evolutionary times, HGT also has a substantial impact on human life, having contributed to the emergence and dissemination of clinical bacteria with multiple resistance to antibiotics, as well as devastating plant pathogens (O'Brien et al., 2012; Martínez, 2018). Comparable accelerated evolution by HGT has also allowed the emergence of new bacterial plant diseases and the distribution of resistance to phytosanitary compounds in agricultural settings (Vivian et al., 2001; Baltrus et al., 2011; Firrao et al., 2018).

Plasmids are the main mobile genetic elements (MGE) driving HGT (Halary et al., 2010), and they can be transferred among distantly-related organisms by conjugation, a process mediating the transfer of DNA and proteins from a donor to a recipient through cell-to-cell contact (de la Cruz et al., 2010; Guzmán-Herrador and Llosa, 2019). Generally, conjugative transfer requires the expression of genes for three separate and specialized systems: (1) a system involved in mating-pair formation (MPF), mediating the physical transfer of DNA from the donor cell to the recipient cell, (2) a DNA transfer and replication (Dtr) delivery system, allowing the specific identification of mobilizable DNA and its delivery to the recipient cell through the MPF system, and (3) a type IV coupling protein, which is an ATPase that couples the two previous systems and is thought to energize the DNA transfer process.

The MPF system involves specialized type IV secretion systems (T4SS), which secrete proteins and nucleoprotein complexes across membranes (Backert and Grohmann, 2017; Rapisarda and Fronzes, 2018). There are eight main classes of MPF systems, class MPF_T_ (previously named type IVA secretion system, or T4ASS) being the most abundant (Guglielmini et al., 2014). The members of these classes are functionally and structurally similar and their constituent proteins can often be exchanged among them. These systems consist of a highly variable number of genes, coding for proteins that assemble into a barrel-like complex spanning the cell envelope and finishing in a conjugation pilus. This pilus makes contact with the recipient cell and serves as a conduit to deliver the DNA as a nucleoprotein adduct.

The main components of the Dtr system are the origin of transfer (*oriT*) and the cognate relaxase, which, together with diverse auxiliary proteins, form the relaxosome. The *oriT* spans no more than one hundred to a few hundred base pairs and contains a few conserved motifs (Zrimec and Lapanje, 2018), specifically identifying the DNA that can be transferred by conjugation. The relaxase is an endonuclease that cleaves the *oriT* in a site- and strand-specific manner forming a covalent bond with its 5′ end, delivering it to the MPF system and piloting the transfer of this single-stranded DNA to the recipient cell. The DNA mobilization region usually contains the *oriT* located in close association with the relaxase gene and a gene for a relaxase accessory protein (RAP). Unlike *oriT* and the relaxase, RAPs are usually not essential for conjugation but increase the mobilization frequency (Backert and Grohmann, 2017; Garcillán-Barcia et al., 2019; Guzmán-Herrador and Llosa, 2019). Relaxases are organized into nine classes, with classes MOB_P_ and MOB_Q_ being prominent in Gammaproteobacteria (Garcillán-Barcia et al., 2020). In general, relaxases are highly specific for their cognate *oriT* sequences, although they can recognize heterologous sequences with a lower efficiency and, with few exceptions, can act both in cis and in trans (Garcillán-Barcia et al., 2019; Guzmán-Herrador and Llosa, 2019). Therefore, mobilizable plasmids are those containing an *oriT*, and generally the cognate RAP and relaxase genes, whereas conjugative plasmids contain both the MPF and the Dtr systems (Smillie et al., 2010).

The plant pathogenic gammaproteobacterium *Pseudomonas syringae sensu lato* (hereafter *P. syringae*) is a taxonomic complex of utmost economic and research importance, causing relevant diseases in nearly all cultivated herbaceous and woody plants (Mansfield et al., 2012). Individual isolates, however, usually have a narrow and distinctive plant host-range that determines their separation into more than 60 pathovars. The rather large pangenome of *P. syringae* (Gomila et al., 2017; Dillon et al., 2019) suggests a very high rate of gene acquisition by horizontal transfer (Brockhurst et al., 2019), which could be mediated by plasmids and/or other MGEs (Sundin and Murillo, 2009; Jackson et al., 2011). Most strains of *P. syringae* usually contain one or more native plasmids that increase bacterial fitness by carrying genes for virulence, conferring resistance to chemicals used in disease control (such as streptomycin or copper), or resistance to UV light, among many others traits. Field and laboratory experiments demonstrated the conjugative transfer of plasmids carrying copper and streptomycin resistance genes among *P. syringae* strains. Additionally, comparative phylogenetic analyses demonstrate an exchange of genes and plasmid fragments within populations and species of *P. syringae* (Vivian et al., 2001; Ma et al., 2007; Sundin and Murillo, 2009). In fact, complete MPF_T_ and MPF_I_ (previously named type IVB secretion system, or T4BSS) systems, as well as genes from both classes, have been found in native plasmids from *P. syringae* (Stavrinides and Guttman, 2004; Zhao et al., 2005; Pérez-Martínez et al., 2008; Gutiérrez-Barranquero et al., 2017). However, neither the functionality of the MPF and Dtr systems and their role in conjugation and mobilization of native plasmids from *P. syringae*, nor the host range of *P. syringae* plasmids have been explored.

*P. syringae* pv. savastanoi NCPPB 3335 causes aerial tumors in olive (*Olea europaea*) plants, and contains three virulence plasmids: pPsv48A (pA, 80 kb), pPsv48B (pB, 45 kb), and pPsv48C (pC, 42 kb). The three plasmids are individually required to induce full symptoms and to reach high populations in planta (Bardaji et al., 2011b; Castañeda-Ojeda et al., 2017a,b; Añorga et al., 2020). Sequencing of the plasmid complement of NCPPB 3335 identified 15 coding sequences (CDSs) that might constitute a complete MPF_T_ in pB, plus a well-conserved putative origin of transfer (*oriT*) in pB and in pC, and an incomplete MPF_I_ system, but not a recognizable *oriT*, in pA (Bardaji et al., 2011b).

Here we demonstrated that the MPF_T_ system of pB participates in the conjugal transfer of pB and pC in solid and liquid media and on plant leaves. The MOB_P_ relaxases of pB and pC were interchangeable, although with differing efficiencies, and we also identified a functional MOB_Q_ mobilization region in pC. pB could be mobilized to diverse phylogroups of *P. syringae*, where they were stably maintained after 21 subcultures.

## Materials and methods

### Bacterial strains, plasmids and growth conditions

Relevant bacterial strains, plasmids and constructions used in this study are listed in Supplementary Table S1. *Pseudomonas* (at 28°C) and *E. coli* (at 37°C) strains were propagated using LB medium (Sambrook et al., 1989), due to the generation of small and round, well-defined colonies on this medium favoring colony counting. Liquid cultures were always incubated with orbital shaking (200 rpm). Counterselection of cells carrying the *sacB* gene, which confers lethality in the presence of sucrose, was done in nutrient agar (NA) medium (Oxoid, Basingstoke, UK) supplemented with 5% sucrose (medium SNA). Conjugations were carried out onto plates of LB, medium B (KMB) (King et al., 1954) or Minimal A Medium (MAM) (Miller, 1992); MAM was routinely supplemented with a final concentration of 0.04 g L^−1^ of casamino acids and 2 g L^−1^ of glucose (MacDonald et al., 1986). When necessary, and unless otherwise indicated, media were supplemented with (final concentrations, in μg mL^−1^): ampicillin (Amp), 100; gentamicin (Gm), 12.5; kanamycin (Km), 7 for *P. syringae* and 25 for *E. coli*; tetracycline (Tc), 3 for *P. syringae* and 12.5 for *E. coli*; spectinomycin (Sp), 25; streptomycin (Sm), 50.

### General molecular procedures and bioinformatics

DNA was amplified using a high fidelity polimerase (PrimeStar HS, Takara Bio Inc., Japan or Q5 High-Fidelity DNA polymerase, New England Biolabs), or a standard enzyme (BIOTaq, Bioline, UK), using primers detailed in Supplementary Table S2, and cloned using the CloneJET PCR Cloning Kit (Thermo Scientific) or the pGEM-T Easy Vector System (Promega). Purification of plasmids from *E. coli* was done following a boiling method (Holmes and Quigley, 1981) or using a commercial kit (Illustra PlasmidPrep Mini Spin Kit, GE Healthcare). For plasmid profile gels, DNA was purified by alkaline lysis and separated by electrophoresis in 0.8% agarose gels with 1xTAE as described (Sesma et al., 2000). Plasmids were transferred to *P. syringae* by electroporation (Choi et al., 2006).

The NCBI BLAST programs (Johnson et al., 2008) and EMBL-EBI (https://www.ebi.ac.uk/services/) server tools (Hubbard et al., 2008) were used to compare and search for homology among sequences and to produce sequence alignments. Alignments were shaded using the Sequence Manipulation suite (Stothard, 2000). BProm (Solovyev and Salamov, 2011) and Mfold (Zuker, 2003) were used to predict binding sites and inverted repeats in putative *oriT* sequences. Search for protein motifs was done using the InterPro interface (Mitchell et al., 2015) (http://www.ebi.ac.uk/interpro/). Genome and nucleotide sequence visualization and manipulation was done using the Artemis genome browser and ACT (Carver et al., 2008). Primers were designed using the Primer3plus software (Untergasser et al., 2012).

Search for T4SSs was done using the web servers for SecReT4 2.0 (https://bioinfo-mml.sjtu.edu.cn/SecReT4/) (Bi et al., 2013) and oriTfinder (https://bioinfo-mml.sjtu.edu.cn/oriTfinder/) (Li et al., 2018), using default parameters. Hits were considered significant with an E-value lower than 10^−5^. We uploaded nucleotide fasta files for all searches because we noticed that, at least for SecReT4, detection was more sensitive in this way than using annotated nucleotide files. To identify relaxes MOB families, the complete set of annotated CDSs of plasmids pA, pB and pC were analyzed with the web server for MOBscan (https://castillo.dicom.unican.es/mobscan/) (Garcillán-Barcia et al., 2020). Search for protein motifs and fold recognition was done using the Pfam (http://pfam.xfam.org/; database version 35.0) (Mistry et al., 2020) and the Phyre2 v 2.0 (Kelley et al., 2015) web servers.

### Construction of mutants

For the construction of mutants by marker-exchange, DNA regions of 0.7–1 kb flanking the target sequence for mutagenesis were selected to insert an antibiotic resistance gene between them and either introduce a deletion or interrupt the gene of interest. The flanking DNA fragments were amplified by PCR using a high-fidelity polimerase and primers containing appropriate restriction sites on both ends (Supplementary Table S2). Amplicons were separately cloned using the CloneJET PCR Cloning Kit (Thermo Scientific) and then cloned in their native orientation into pK18*mobsacB* (Km^R^) or pJQ200SK (Gm^R^) as a single fragment, with an appropriate antibiotic resistance gene separating the two fragments. For this, we used the Km^R^ cassette from pK18*mobsacB* (Schäfer et al., 1994), the Gm^R^ cassette from pJQ200SK (Quandt and Hynes, 1993), the Sm^R^/Sp^R^ cassette from pHP45Ω (Prentki and Krisch, 1984), or the Tc^R^ cassette from pHP45Ω-Tc (Fellay et al., 1987), the last two of which introduce transcriptional stops and/or translational stops in all reading frames. The constructs were transferred to *P. syringae* by either conjugation or electroporation, and marker exchange clones were selected as being resistant to sucrose and sensitive to the antibiotic resistance conferred by the vector. All mutations were confirmed by PCR and sequencing. The recipient strain ΔABCTc contains a deletion of 223 nt (positions 1216031..1216253 in accession no. NZ_CP008742) into the pseudogene PSA3335_RS06050 that was replaced by the Tc^R^ cassette from pHP45Ω-Tc. Other mutations are described in Supplementary Table S1.

Transposon Tn*5*-GDYN1 confers diverse antibiotic resistances and sensitivity to sucrose (Supplementary Table S1), mediated by gene *sacB*, allowing for the selection of derivatives cured of tagged plasmids by selection on media with sucrose (Flores et al., 1993). Mutagenesis with this transposon, selection of tagged plasmids and identification of the insertion point were done as described previously (Bardaji et al., 2019).

### Conjugation conditions

Conditions for conjugation are as detailed below. In all cases, except when indicated, experiments were repeated at least three times each with four technical replicates, and means were compared by an analysis of variance (one-way ANOVA *p* > 0.05), followed by Duncan's multiple range test (*p* < 0.05). For the estimation of transfer frequencies, the detection limit was around 10^−7^ transconjugants per recipient. Routinely, we confirmed the transfer of plasmids by examining the plasmid profile by gel electrophoresis of at least 10 randomly chosen transconjugants.

#### Transfer on solid media

For a simple conjugation protocol, cells from liquid cultures in LB grown overnight with selecting antibiotics were collected, washed with one volume of ¼ strength Ringer's solution (¼ Ringer's; Oxoid, Basingstoke, UK) and adjusted to an optical density at 600 nm (OD_600_) of 1 in this buffer. Frequencies of transfer using this protocol could be very high, but were highly variable among experiments. For a standardized conjugation protocol, producing repetitive conjugation transfer frequencies, cells grown overnight in liquid LB with antibiotic selection were diluted in the same medium to an OD_600_ of 0.1, and incubated until they reached an OD_600_ of 0.25. Cultures were then collected, washed and resuspended in the same volume of ¼ Ringer's. After this, we proceed equally for both protocols. Equal volumes of suspensions of donor and recipient strains were mixed and 10 μL were deposited onto plates of LB, KMB or MAM, dried and incubated at 28°C. Each 10 μL spot was individually collected after 24 h (LB and KMB) or 48 h (MAM) and resuspended in ¼ Ringer's; then, 25 μL from 10-fold serial dilutions were spotted onto LB plates with appropriate antibiotics to estimate the transfer frequency. All liquid cultures were incubated at 28°C with shaking (200 rpm).

To evaluate the transfer of native plasmids to other pseudomonads, we used as donor strain FAM-098 (Supplementary Table S1), an arginine auxotroph derivative of strain NCPPB 3335, transformed with either pB::Tn*5*-GDYN1 or pC::Tn*5*-GDYN1. A loopful each of donor and recipient bacteria grown on LB plates overnight were mixed in 250 μL of ¼ Ringer's, and 10 μL of the suspension spotted onto MAM medium. After 48 h at 28°C, the conjugation mix was stripped onto the minimal medium SSM (Meyer and Abdallah, 1978) plus kanamycin to select for transconjugants. The transfer experiments were repeated twice, each with four technical replicates. Transfer of pB and pC was confirmed by PCR and the examination of the plasmid profile of at least two transconjugants per recipient.

#### Transfer on liquid media

Cells from liquid cultures in LB grown overnight with selecting antibiotics were collected, washed with one volume of ¼ Ringer's (Oxoid, Basingstoke, UK) and adjusted to an OD_600_ of 1 in this buffer. Suspensions containing equal volumes of donor and recipient strains were diluted 1:10 in liquid medium LB, KMB or MAM and grown in the dark at 28°C with shaking (200 rpm) until reaching an OD_600_ of 1–1.5. Then, transfer frequency was estimated from population counts by spotting 25 μL of serial 10-fold dilutions in ¼ Ringer's onto LB plus appropriate antibiotic selection.

#### Transfer on plant surfaces

Bean plants (*Phaseolus vulgaris* cv. Canadian Wonder) were grown in peat/perlite (2:1) until the primary leaves were fully expanded (around 10 d), in a growth chamber with a 16/8 h (light/dark) photoperiod at 22/18°C. Strains NCPPB 3335 and ΔABCTc were scraped off LB plates incubated overnight at 28°C, washed and resuspended in 10 mM MgCl_2_ to an OD_600_ of 0.1 (ca. 10^7^ cfu mL^−1^). A 1:1 mix of both strains was sprayed onto both sides of primary leaves using an airbrush (VL Double Action; Paasche Airbrush Company, Kenosha, WI, USA) with an air pressure of 1 bar, until runoff; an experimental replica consisted of two inoculated plants and the experiment was repeated three times. Five days after inoculation, 2 leaf discs were excised per plant with a No. 4 cork borer (0.6 cm diameter), homogenized in 1 mL of ¼ Ringer's and serial dilutions plated onto LB with or without antibiotic selection for population counts.

### Plasmid stability assays

The stability of pB in relation with its conjugative ability was estimated using strain ΔABC containing either pB::Tn*5*-GDYN1 or the non-conjugative variant pB(*virB4*::Km-*sacB*), containing a Km^R^-*sacB* cassette inactivating gene *virB4*. Overnight cultures of these strains on MAM plates were used to prepare suspensions adjusted to an OD_600_ of 0.1 (ca. 10^7^ cfu mL^−1^), and 20 μL were deposited onto MAM plates. After 24 h incubation at 28°C, the bacterial growth was scraped off the plates and resuspended in 1 mL of liquid MAM, and 20 μL of these suspensions was deposited onto MAM plates. The strains were serially transferred in this way for a total of four transfers, and plasmid loss was examined from the starting cultures and after the first and fourth transfer. For this, serial dilutions of the bacterial suspensions were plated onto NA and 100 well-isolated colonies for each strain and time point were replica-plated onto NA, NA+Km, and SNA. The proportion of clones that had lost plasmid pB was estimated from the fraction of clones that were resistant to sucrose and sensitive to kanamycin. Loss of pB was confirmed in random samples of clones by plasmid profile analysis. The experiment was repeated three times with three replicates.

We tested long-term plasmid stability of plasmid pB on different bacteria as described previously (De Gelder et al., 2007; Bardaji et al., 2019). Newly obtained transconjugants of *P. syringae* pv. phaseolicola 1448A and *P. viridiflava* CECT458 containing pB::Tn*5*-GDYN1, which were maintained at −80°C, were grown overnight on LB plus kanamycin, and a single colony was used to start a culture in 3 mL of LB. After overnight growth, 10 μL of this culture were transferred to 3 mL of LB and incubated in the same conditions for 24 h. We estimated populations of *ca*. 10^7^ cfu in the initial inoculum and of *ca*. 10^9^ cfu after 24 h of incubation; therefore, we estimated that ~7 generations were obtained per growth cycle. After 21 serial transfers in this way, with a conservative estimate of at least 150 generations, cultures were plated on LB and 100 well-isolated colonies were replica-plated on LB plates with and without kanamycin, to estimate the proportion of plasmid-free cells. The experiment was repeated three independent times, each with four technical replicates. In each assay, we examined the plasmid profile of at least 10 random Km^R^ colonies to ensure that the plasmids had not undergone any structural changes or become integrated in the chromosome.

### Accession numbers

The accession numbers of the relaxases investigated here are: for pB, UniProtKB H1ZX26 (MobB, MOB_P_ family); and for pC, UniProtKB H1ZX53 (MobB, MOB_P_ family) and UniProtKB H1ZX69 (TraA, MOB_Q_ family).

## Results

### Bioinformatics identification of conjugation genes

**Figure 1:**
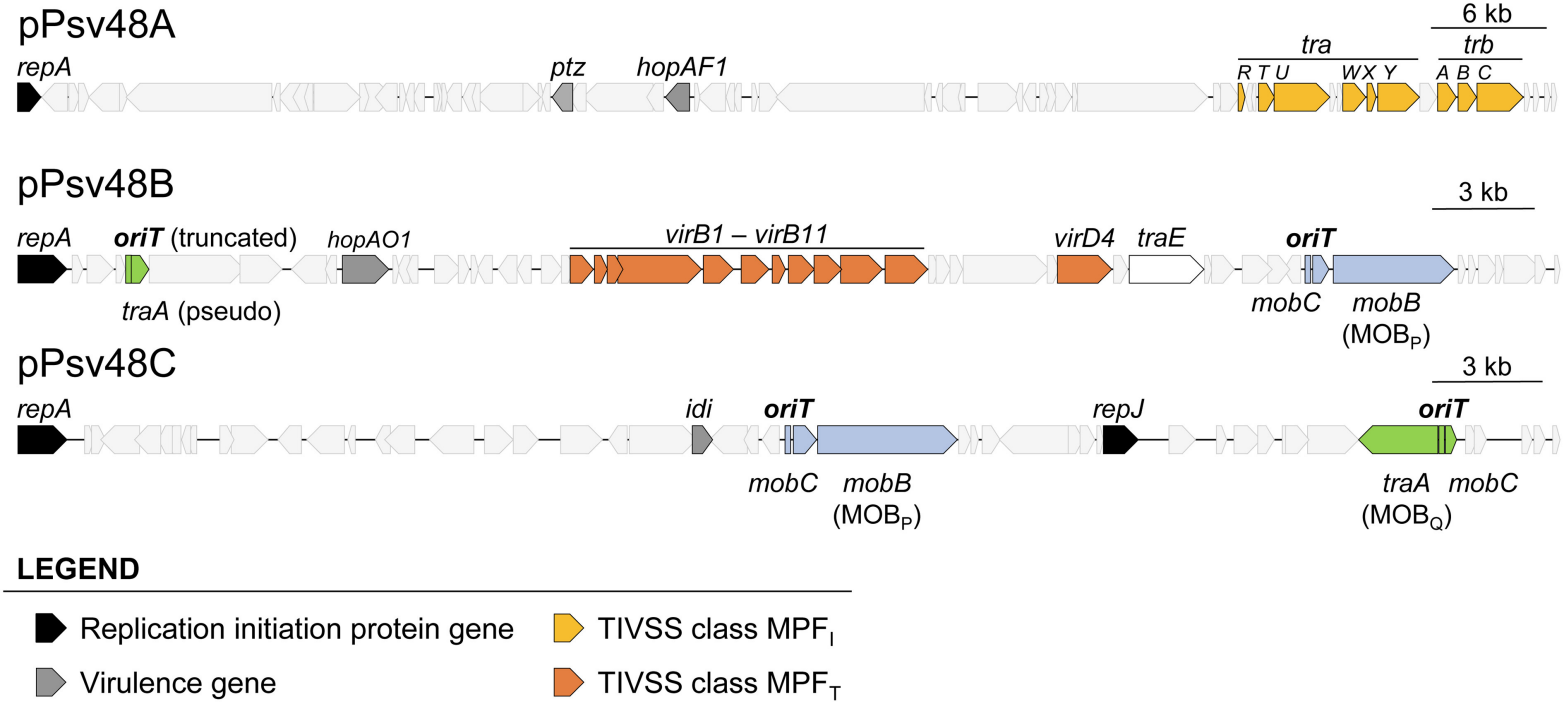
Relevant genes in the three virulence plasmids of *Pseudomonas syringae* pv. savastanoi NCPPB3335. Location and extent of genes involved in replication, virulence, synthesis of type IV secretion systems, and relaxases and associated RAPs. The location and extent of the origin of transfer associated to the *traA* genes is speculative and based on other members of the MOB_Q_ family relaxases.

The sequences of plasmids A, B and C were analyzed with the software SecReT4 and oriTfinder, as well as by manual BLAST, allowing us to refine the previous annotation of genes involved in conjugation (Bardaji et al., 2011b), eliminating misidentifications and identifying overlooked gene fragments (Figure 1, Supplementary Table S3). As previously described (Bardaji et al., 2011b), we confirmed that pA contains a partial set of MPF_I_ genes whereas pB contains a complete MPF_T_ system (Supplementary Table S3). Furthermore, pB contains a gene coding for a putative type IA topoisomerase homologous to TraE from plasmid RP4 (Supplementary Table S3), which could participate in resolution of cointegrates during vegetative or conjugative replication (Li et al., 1997).

For the identification and classification of relaxases in the three virulence plasmids, we used the two previous programs and the MOBscan software (Figure 1, Supplementary Table S3), which offered partial and conflicting results. Alleles of gene *mobB* are present in both pB and pC (Figure 1, Supplementary Figure S1), and MOBscan identified their deduced products as relaxases of the MOB_P_ family. Remarkably, SecReT4 and oriTfinder identified the *mobB* gene from pB as a *virB8* conjugation gene but did not report the *mobB* gene from pC, despite the high similarity of the MobB products (86.6% aa identity; Supplementary Figure S1). A Pfam search of the MobB products found a Relaxase/Mobilization nuclease domain (PF03432), which Pfam reported significant for pC (E value 1.0e-6) but not for pB (E value 7.8e-3), and a significant (E value ≤ 1.7e-14) Large polyvalent protein-associated domain (PF18821), which is widely present in proteins transmitted by conjugation (Iyer et al., 2017). The structure of the N-terminal residues of both MobB products could be modeled by Phyre2 with more than 90% confidence, being similar to diverse relaxase domains and a replication initiator protein. This is not surprising because it is described that relaxases share motifs with replication proteins (Guglielmini et al., 2014; Guzmán-Herrador and Llosa, 2019). We therefore considered that the *mobB* genes from pB and pC coded for bona fide relaxases of the MOB_P_ family.

The three programs also predicted a second relaxase gene, gene *traA*, in both plasmids, coding for a MOB_Q_ family relaxase, except that the gene in pB (PSA3335_RS00025) is truncated and likely not functional (Supplementary Figure S1, Supplementary Table S3).

oriTfinder identified the *oriT* sequences accompanying the MOB_P_ relaxase genes in pB and pC but not a putative *oriT* associated to the MOB_Q_ relaxase genes (Supplementary Figure S2). None of the programs identified any of the *mobC* RAP genes accompanying the relaxase genes; at least for the MOB_P_ RAPs this could be due to their low sequence conservation (Supplementary Figure S1B). No relaxase genes nor *oriT* were found in plasmid pA.

Previous research demonstrated the chromosomal integration of native plasmids in *P. syringae* strains (Szabo and Mills, 1984; Neale et al., 2013). We therefore examined the possible presence of relaxases in the chromosome of strain NCPPB 3335, where we have previously found remnants of plasmid genomes (J. Murillo and C. Ramos, unpublished data). A blastp search using the pB and pC relaxases as query against the genome of strain NCPPB 3335 (accession no. NZ_CP008742), as well as a simple text search of its annotation, identified four CDSs annotated as relaxases or TraI domain-containing protein, which were examined with MOBscan. Two of them, PSA3335_RS05310 and PSA3335_RS09015, belong to the MOB_P_ family and are preceded by a RAP gene. In blastp comparisons, PSA3335_RS05310 and PSA3335_RS09015 showed 30% identity with around 80% coverage, whereas only PSA3335_RS09015 showed detectable sequence homology with the MOB_P_ relaxases from plasmids B and C, returning alignments with around 29% identity over 157 amino acids or less. CDS PSA3335_RS07700 is a pseudogene and PSA3335_RS23785 belongs to the MOB_H_ family and codes for a putative relaxase from an integrating conjugative element.

### Two virulence plasmids are mobilizable

We evaluated the conjugational transfer of the three virulence plasmids from strain NCPPB 3335 using diverse strains as donors. These strains contained combinations of one to three plasmids with only one of them tagged with transposon Tn*5*-GDYN1 (Km^R^; Supplementary Table S1). As the recipient, we routinely used strain ΔABCTc, a derivative of strain NCPPB 3335 cured of the three plasmids and tagged with a tetracycline-resistance (Tc^R^) cassette (Supplementary Table S1). Using a simple conjugation protocol, we initially observed conjugation frequencies of pB as high as 3.7 ± 0.8 × 10^−1^ transconjugants per recipient, but with experiment-to-experiment variations of two orders of magnitude or more. We therefore used a standardized conjugation protocol (see materials and methods), which resulted in lower conjugation frequencies but provided consistent results in different experiments.

**Figure 2:**
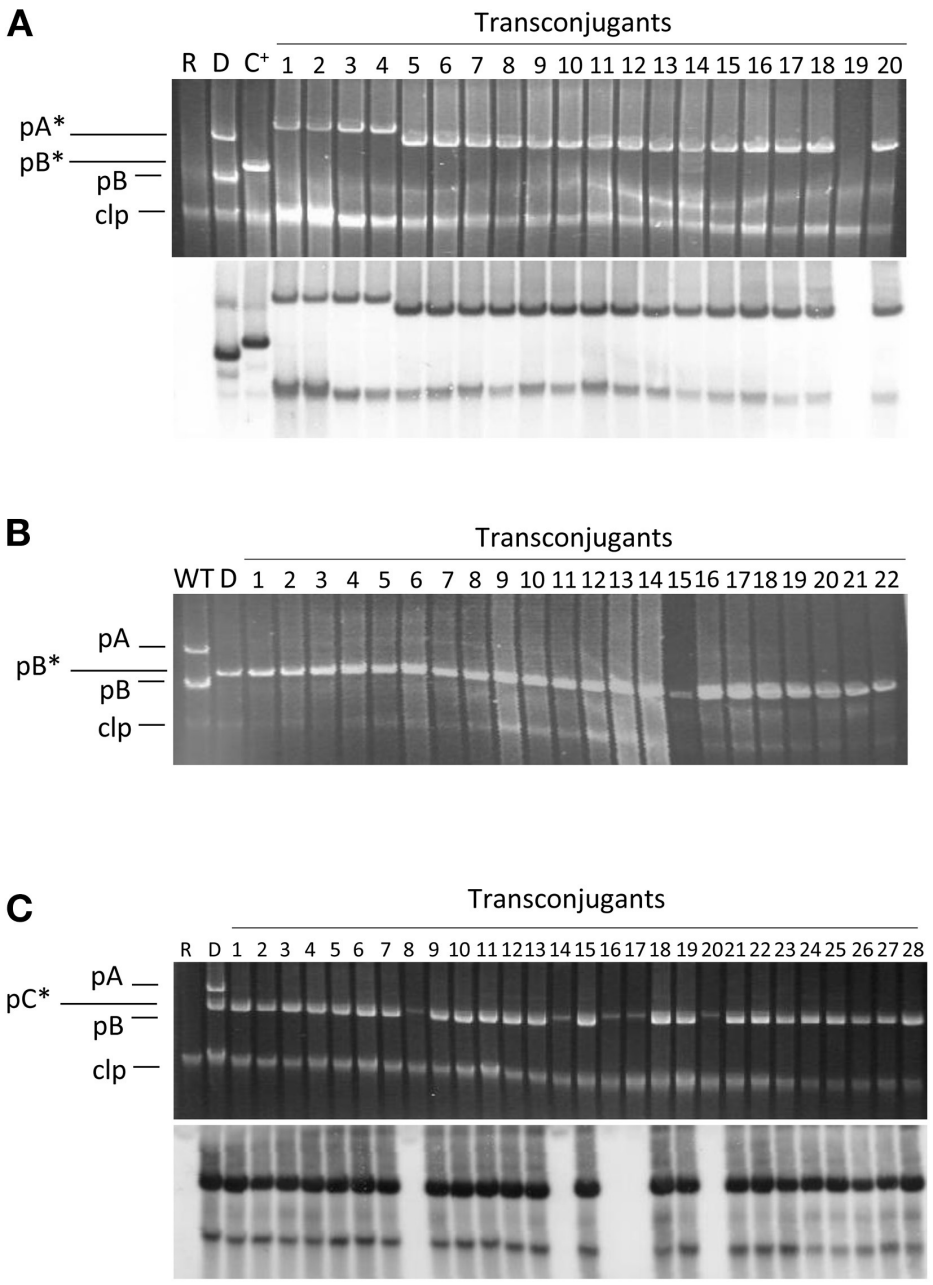
Plasmid profiles of transconjugants and their hybridization to a probe specific for pB. Conjugation was carried out on solid minimal medium A using the plasmidless strain ΔABCTc as recipient and as donors the following strains: **(A)** strain NCPPB 3335 containing pPsv48A::Tn*5-*GDYN1 (pA*); **(B)** strain ΔABC containing pPsv48B::Tn*5-*GDYN1 (pB*); **(C)** strain NCPPB 3335 containing pPsv48C::Tn*5-*GDYN1 (pC*). The bottom picture in **(A,C)** correspond to the gels shown above after being subjected to Southern hybridization using as probe a region from pB (positions 14062..15310, accession no. FR820586) not repeated elsewhere in the genome of strain NCPPB 3335. WT, wild type strain NCPPB 3335; D, donor strain; R, receptor strain ΔABCTc; C+, hybridization control (strain ΔABC with pB*); clp, chromosome and linear plasmids.

In standard mating experiments on solid media, we did not detect the transfer of pA (Table 1). However, using donors with a tagged pA in matings with the simple conjugation protocol, which allows for higher transfer frequencies, we occasionally obtained one or two Tc^R^-Km^R^ transconjugants per mating (transfer frequency ≤ 0.8 ± 0.2 × 10^−6^ transconjugants per recipient). We examined the plasmid profiles of 20 randomly chosen transconjugants to confirm plasmid transfer (Figure 2). All clones suspected to have received pA contained a plasmid of similar or larger size than pA but that hybridized to a pB-specific probe (Figure 2). One of the clones was apparently plasmidless and did not hybridize with the pB probe; we therefore assumed that during the mating process this clone had received a copy of Tn*5*-GDYN1, which is functional for transposition. By PCR with specific primers, we determined that four of these 20 clones contained both virulence genes *ptz* (from pA) and *hopAO1* (from pB), whereas some other clones contained assorted combinations of maintenance genes from pA and pB (data not shown). This suggests that chimeric molecules between plasmids pA and pB might be formed and subsequently mobilized with very low frequency, and that pA is not mobilizable in its native form. Therefore, we did not examine the transfer of pA in any further experiments.

**Table 1:** Frequency of transfer of virulence plasmids from *P. syringae* pv. savastanoi NCPPB 3335 in solid and liquid media.

	**Frequency of transfer for each plasmid (**× **10**^**−6**^**) per donor strain^a^**					
	**NCPPB 3335**			**Psv48**Δ**A**		**ΔABC**
**Medium**	**pA**	**pB**	**pC**	**pB**	**pC**	**pB**
**Solid media**						
KMB	bdl^b^	5,000 ± 200	1.3 ± 0.2	nd	nd	2,700 ± 100
MAM	bdl	9,500 ± 1,300	280 ± 100	15,000 ± 1,000	1,200 ± 300	14,500 ± 1,290
**Liquid media**						
LB	nd^c^	bdl	bdl	nd	nd	nd
KMB	nd	4.7 ± 4.5	bdl	nd	nd	nd
MAM	nd	13 ± 10	5.1 ± 0.9	nd	nd	nd

a Frequency of transfer (number of transconjugants per recipient) of virulence plasmids from *P. syringae* pv. savastanoi NCPPB 3335 derivatives to the plasmidless strain ΔABCTc, in solid and liquid media. The donor strain contained one to three plasmids, but only one in each case tagged with Tn5-GDYN1.

b bdl, below the detection limit. In our conditions, the limit was around 10^−7^ transconjugants per recipient.

c nd, not determined.

Conversely, we routinely obtained Tc^R^-Km^R^ transconjugants with tagged variants of pB and pC (Table 1), indicating that these native plasmids can be mobilized. The frequency of conjugation was high for pB, reaching over 10^−2^ transconjugants per recipient, whereas the transfer frequency of pC was always at least one order of magnitude lower. Transfer of pC only occurred when the donor also contained pB (Table 1 and data not shown). This indicates that pB contains all the machinery for its conjugative transfer, whereas pC is mobilizable.

Plasmid transfer occurred both on solid and in liquid media, although frequency of transfer was significantly higher on solid media (Table 1). Additionally, transfer was highest on minimal medium (MAM), whereas on rich media transfer was consistently higher on KMB than on LB (Table 1 and data not shown). Therefore, all matings were hereafter carried out on MAM plates.

We routinely examined the plasmid profiles of selected transconjugants to confirm plasmid transfer. All examined transconjugants from matings with the tagged plasmids B and C contained the plasmid band of the corresponding size, indicating that these plasmids can be efficiently mobilized (Figure 2). However, a sizable portion of the transconjugants selected for the acquisition of pC::Tn*5*-GDYN1 also contained pB. To estimate the frequency of co-transfer, we used as donor strain NCPPB 3335 (pC::Tn*5*-GDYN1) (Km^R^) and the plasmidless strain ΔABCTc (Tc^R^) as recipient in mating experiments. The plasmid profiles of 300 Km^R^-Tc^R^ transconjugants from three independent experiments were analyzed visually and by Southern hybridization with a probe specific for plasmid pB (Figure 2 and data not shown). This showed that an average of 80% of the transconjugants had also acquired pB, indicating a frequent co-transfer of plasmid pB when pC is mobilized. Taking into account the data in Table 1, this may also imply that when pB is transferred by conjugation, pC is also transferred between 2 and 6% of the time.

### The predicted *oriT* from pB and pC associated to MOB_P_ relaxases are functional

Our previous annotation (Bardaji et al., 2011b) and the software oriTfinder predicted the presence of an *oriT* associated to the MOB_P_-family relaxase gene in both pB (107 nt) and pC (146 nt) (Supplementary Figure S2). To evaluate their functionality, fragments of 221 (pB) or 220 nt (pC) containing the predicted *oriT* (Supplementary Figure S2) were cloned into the non-mobilizable plasmid pKMAGJ and electroporated into strain NCPPB 3335. Both cloned fragments were transferred to strain ΔABCTc with average frequencies of 2.2 ± 1.5 × 10^−5^, for *oriT*-pB, and 3.3 ± 0.4 × 10^−4^ transconjugants per recipient for *oriT*-pC. These results indicate that the cloned sequences contain functional *oriT* sequences. Their lower transfer frequency compared to those of the whole plasmids (Table 1) suggest that (1) they might be missing sequences important for the transfer process, (2) that they compete inefficiently for the relaxase with the native copy of the *oriT*, and/or (3) that the MobB relaxases might act more efficiently in cis for their cognate *oriT*.

Despite repeated efforts, we were not able to clone the *oriT* associated to *traA* in pC for functional testing.

### The MPF_T_ from pB is essential for the mobilization of the virulence plasmids

Gene *virB4* is part of the MPF_T_ from pB and codes for a highly conserved protein widely present in different MPF clusters (a cytoplasmic ATPase providing energy for pilus assembly), thus being essential for conjugation (Peña et al., 2012). We constructed a derivative of pB containing a Gm-resistance cassette replacing the last 1092 nt of the *virB4* CDS, causing the deletion of 42.8% of the C-terminal end of the VirB4 protein, resulting in plasmid pB(*virB4*::Gm) (Supplementary Tables S1, S2). Nevertheless, and since *virB4* is part of a large operon, it is feasible that this mutation could exert polar effects and alter the expression of other downstream genes involved in conjugation.

In mating experiments with strain ΔABCTc as recipient, a strain containing pB(*virB4*::Gm) did not transfer either this plasmid or pC::*sacB* at detectable levels. These results then indicate that the mobilization of the virulence plasmids of strains NCPPB 3335 depends entirely on the functionality of the MPF_T_ from pB.

### Mobilization of pB does not appear to contribute to plasmid stability

Low-copy number plasmids generally contain diverse maintenance determinants ensuring their stable vertical inheritance (plasmid stability) (Nordström and Austin, 1989). However, our previous analyses did not find any relevant maintenance determinants in pB (Bardaji et al., 2019). Therefore, we evaluated the possibility that conjugative transfer might contribute to the stable inheritance of pB, as was described for other plasmids (De Gelder et al., 2007). Since the *sacB* gene causes lethality in the presence of sucrose, we could estimate the loss rate of pB::Tn*5*-GDYN1 or of the non-conjugative variant pB(*virB4*::Km-*sacB*), both containing *sacB*, from the proportion of sucrose-resistant colonies growing on sucrose-containing media, as previously described (Flores et al., 1993; Bardaji et al., 2019). To avoid false negatives, from counting those cells containing a plasmid with a spontaneous mutation inactivating gene *sacB*, we only considered that the plasmid was lost when the cells were resistant to sucrose (Suc^R^) and sensitive to kanamycin (Km^S^). To also eliminate any possible interference, we evaluated the loss of pB in strains containing only this plasmid.

**Figure 3:**
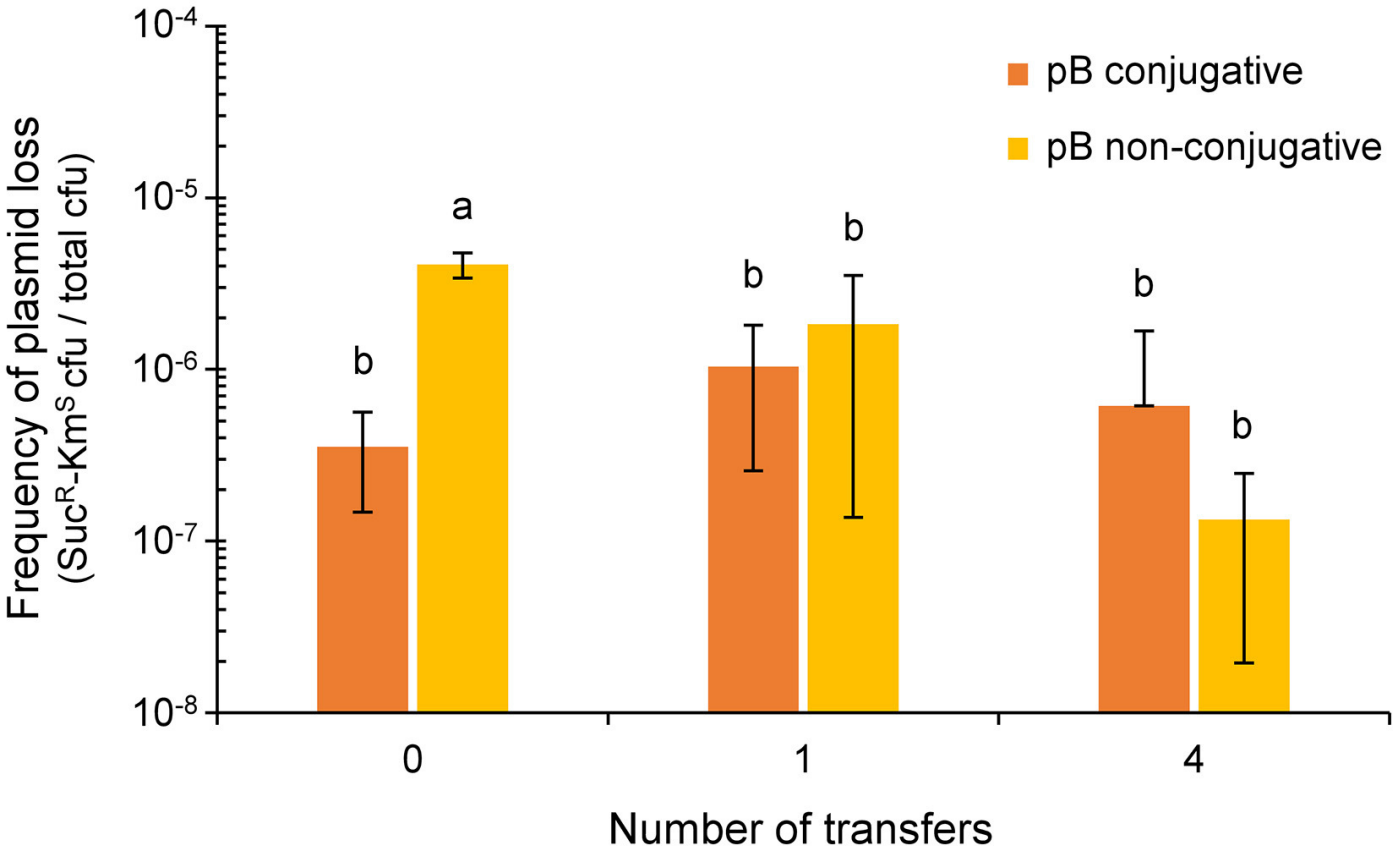
Effect of the conjugative ability of pB (pPsv48B) on plasmid loss. Colored bars indicate the frequency of plasmid loss (mean ± sd), measured as the number of sucrose-resistant and kanamycin-sensitive colonies per total colony forming units in unsupplemented medium. The plasmidless strain *P. syringae* pv. savastanoi ΔABC containing tagged derivatives of pB that were either conjugative (pB::Tn*5*-GDYN1, dark orange) or non-conjugative (pB[*virB4*::Km-*sacB*], light orange) were subcultured every 24 h on solid medium MAM. Values are the average of at least three independent experiments, each with three replicates. Means with different letters are significantly different (one-way ANOVA and Duncan's multiple range test; *p* < 0.05).

After one day of growth on solid MAM, the fraction of Suc^R^-Km^S^ cells (around 10^−6^ cfu mL^−1^; Figure 3) was not significantly different between the cultures containing pB::Tn*5*-GDYN1 or of the non-conjugative variant pB(*virB4*::Km-*sacB*). We obtained similar results after 4 days of daily transfers of each culture on solid fresh medium (Figure 3). In our particular experimental conditions, therefore, the conjugative ability of pB does not significantly contribute to the maintenance of this plasmid in the bacterial population.

### Plasmids pB and pC can be mobilized by two to three different relaxases

**Figure 4:**
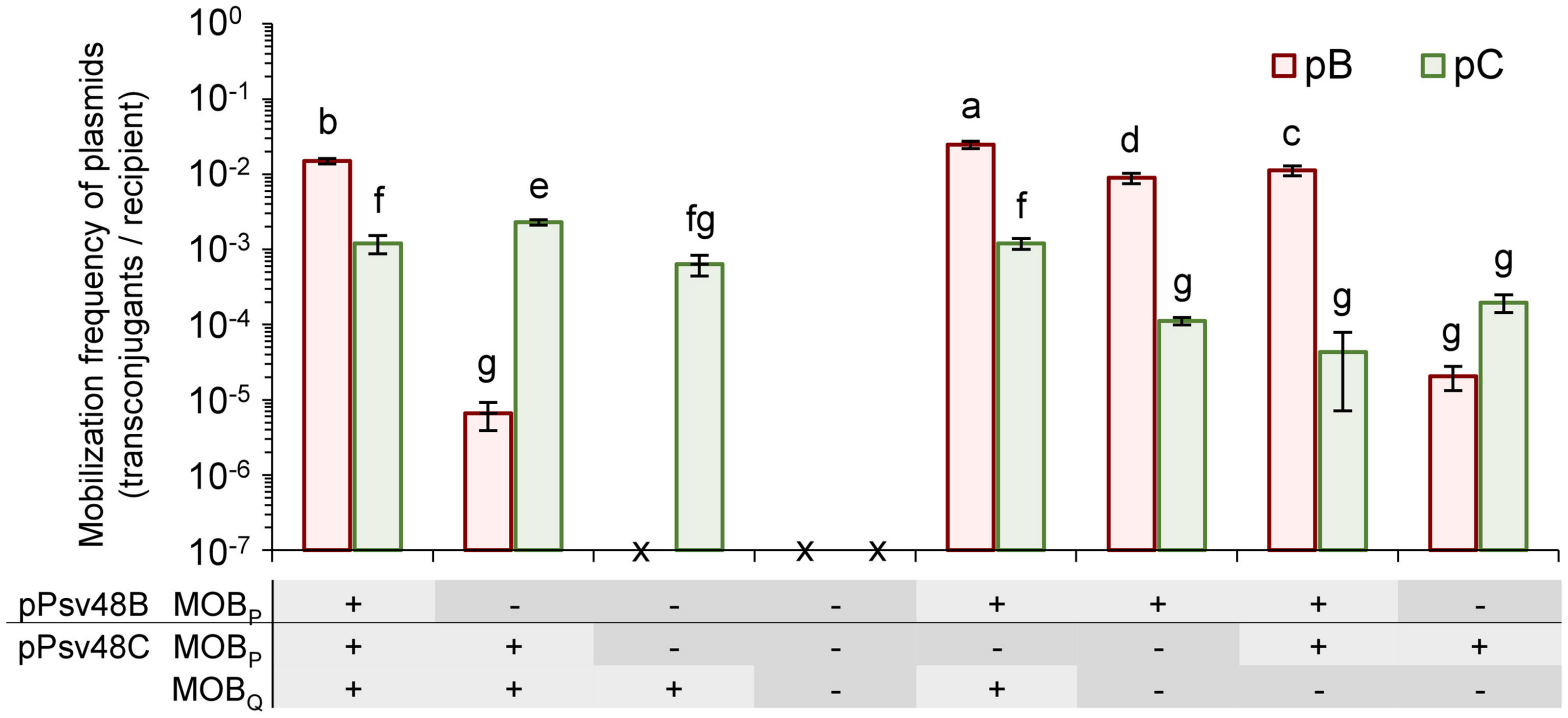
Role of the three relaxase genes in the conjugal mobilization of pB and pC evidenced by site-directed mutagenesis. Bars indicate the mobilization frequency (mean ± sd) of plasmids pB (orange) and pC (green) in strains containing functional (+) or mutated (–) versions of the three relaxase genes, as indicated in the panel below the graph. x, no transconjugants detected. Values are the average of at least three independent experiments, each with four replicates. Means with different letters are significantly different (one-way ANOVA and Duncan's multiple range test; *p* < 0.05).

Plasmids pB and pC each contain a MOB_P_ relaxase gene, whereas pC contains an additional full-length MOB_Q_ relaxase gene. To explore the role of these three relaxases in conjugative transfer, we constructed diverse strains containing insertions of an antibiotic resistance cassette in one or more of the three relaxase genes. As donors in mating experiments we used strains containing plasmids pB and pC, with appropriate relaxase mutations, and the plasmidless strain ΔABCTc as a recipient (Figure 4).

The two MOB_P_ relaxases were interchangeable and could mobilize both plasmids pB and pC (Figure 4). Plasmid pC was mobilized with the same low frequency by both relaxases; however, they were not equally efficient for the mobilization of pB, with a drop of at least two orders of magnitude in the frequency of transfer with the relaxase from pC (Figure 4).

The MOB_Q_ relaxase mobilized only plasmid pC, and with a frequency of transfer that was not significantly different to those of the MOB_P_ relaxases. The lack of mobilization of pB could be due to the fact that pB contains only a partial putative *oriT* associated to its truncated MOB_Q_ relaxase gene (Supplementary Figure S2). Additionally, it is likely that the MOB_Q_ relaxase show a cis-acting preference, as is typical of this protein family (Garcillán-Barcia et al., 2019), which could reduce the potential frequency of transfer of pB to undetectable levels.

Remarkably, the coexistence of different relaxases had a subtle but significant effect, with different combinations of functional relaxases increasing or reducing the transfer frequency of the individual plasmids (Figure 4). For instance, the frequency of transfer of pC was maximized with only two functional relaxase genes when the MOB_P_ gene in pB was mutated.

Finally, we could not detect the mobilization of plasmids B or C in strains containing mutated versions of the three relaxase genes (Figure 4), even if the donor strain also contained an intact copy of plasmid pA (data not shown). This data indicates that the conjugative transfer of plasmids B and C is exclusively mediated by their own relaxase genes.

### Plasmid pB also transfers among epiphytic populations

We wanted to evaluate the possibility that the virulence plasmids of NCPPB 3335 could be transferred among bacteria on plant surfaces. We decided to use bean plants for these experiments because of the advantages of using a herbaceous plant and because bean is a non-host plant for strain NCPPB 3335; additionally, strain NCPPB 3335 is phylogenetically close to different pathovars infecting bean.

To test this, we spray inoculated bacterial suspensions containing a 1:1 mixture (~2.5 × 10^7^ cfu mL^−1^ each) of strains ΔABC(pB::Tn*5*-GDYN1) and ΔABCTc onto bean leaves. The strains survived only poorly on bean leaves, and at 5 dpi we could find bacterial populations only in two out of the six inoculated plants, ranging from 0.05 × 10^−2^ to 3 × 10^−2^ cfu leaf disc^−1^. Nevertheless, in these plants we observed the transfer of pB at a frequency of around 2 × 10^−2^ transconjugants per recipient, demonstrating that plasmid pB can be transferred in natural epiphytic populations. Since bean is a non-host plant, and because *P. syringae* strains can persist on many different plants and other reservoirs (Morris et al., 2019), this suggests that strain NCPPB 3335 could potentially transfer its virulence plasmids to a wide bacterial population in different environments.

### pB mobilizes to different bacteria

Plasmids, like other MGEs, are usually distributed preferentially to phylogenetically related bacteria (Smillie et al., 2010; Jackson et al., 2011; Bardaji et al., 2017b). To test the transfer of pB to other bacteria, we transformed pB::Tn*5*-GDYN1 in an NCPPB 3335 derivative auxotrophic for arginine, strain FAM-098 (Matas et al., 2012), to be used as donor. As recipients, we tested 46 strains from eight phylogroups of *P. syringae* (Dillon et al., 2019) and other *Pseudomonas* species, and transconjugants were selected on SSM plus kanamycin.

Plasmid pB was efficiently transferred to 22 strains belonging to six phylogroups of *P. syringae* (Table 2 and Supplementary Table S4). However, we did not detect its transfer to any of the remaining strains tested. We evaluated the stability of pB in two recipient strains chosen as examples, *P. syringae* pv. phaseolicola 1448A and *P. viridiflava* CECT458. After 21 serial transfers on liquid LB medium, where pB is not transferred by conjugation (Table 1), we did not observe the generation of plasmid-free segregants. These results indicate that pB can potentially be distributed widely at least within *P. syringae*, and that it would be stably maintained once acquired.

**Table 2:** Conjugative transfer of plasmids pB and pC from *P. syringae* pv. savastanoi NCPPB 3335 to different strains and species of *Pseudomonas*^a^.

	**Acceptors/total** ^b^	
***Pseudomonas*** **spp**.	**pB**	**pC**
*P. marginalis*	0/1	–
*P. putida*	0/1	–
*P*.*syringaesensulato*^c^		
1 (3, 8)	3/9	0/4
2 (1)	3/6	0/3
3 (2)	12/19	2/9
4 (4)	1/1	–
5 (9)	0/2	0/1
6 (7)	2/2	–
7 (6)	1/3	0/2
11	0/1	–
uk	0/1	0/1
Total	22/46	2/20

a See Supplementary Table S4 for full details.

b Number of strains accepting plasmid pB or pC/total number of strains assayed; –, not assayed.

c Phylogroups (genomospecies), as described previously (Gardan et al., 1999; Bull and Koike, 2015; Gomila et al., 2017; Dillon et al., 2019); uk, unknown phylogroup and genomospecies.

Conversely, we observed the transfer of pC to only two strains out of the 20 tested, and both from the same phylogroup that includes the plasmid parent strain *P. syringae* pv. savastanoi NCPPB 3335.

## Discussion

*P. syringae* pv. savastanoi NCPPB 3335 contains three virulence plasmids: plasmid pA contains the cytokinin biosynthesis gene *ptz* and the type III effector *hopAF1*, pB contains the type III effector gene *hopAO1*, and pC carries the cytokinin biosynthesis gene *idi* (Bardaji et al., 2011b; Castañeda-Ojeda et al., 2017b; Añorga et al., 2020). Here we showed that the two small virulence plasmids, pB and pC, are transferable horizontally by conjugation in free living conditions, both in solid and liquid media, and on epiphytic populations of a non-host plant. Additionally, we observed that fragments of the large virulence plasmid pA can also be transferred at very low frequency after being cointegrated with plasmid pB. Therefore, the plasmid-borne virulence genes of *P. syringae* pv. savastanoi can be efficiently transferred horizontally by conjugation.

As occurs with other *P. syringae* strains (Zhao et al., 2005), native plasmids of strain NCPPB 3335 contained a variety of MPF systems, with an incomplete version of an MPF_I_ (in pA) and a complete MPF_T_ (in pB). Not surprisingly, the incomplete system of plasmid pA appears to be non-functional and mobilization of all plasmids of strain NCPPB 3335 was dependent on the complete MPF_T_ system of pB. The evaluation of specific knockout mutants (Figure 4) also showed that the MPF_T_ system and the T4C protein VirD4 from pB are promiscuous, being able to efficiently recognize both the MOB_P_ and MOB_Q_ relaxosomes. This is important, because not all MPF_T_ systems can mobilize plasmids with MOB_Q_ relaxases (Garcillán-Barcia et al., 2019), and suggests that these plasmids have undergone selection for the promiscuous use of conjugation systems to maximize their chances for horizontal distribution.

The native plasmids of strain NCPPB 3335 contained three functional plasmid-borne relaxase genes (Figures 1, 4), one located in pB and two in pC. The MOB_P_ relaxases found in pB and pC were functionally interchangeable, although with differing efficiency that was determined both by the relaxase and the *oriT* (Figure 4). Thus, pB was transferred with high frequency by its own MOB_P_ relaxase but with a frequency at least two orders of magnitude lower by the MOB_P_ relaxase from pC, suggesting a high specificity with its cognate *oriT*. Conversely, pC was always transferred with low frequency by both MOB_P_ relaxases. The third relaxase, from the MOB_Q_ family, only allowed the transfer of pC, which was expected because pB did not appear to contain a cognate functional origin of transfer. Remarkably, our data indicate that different combinations of functional relaxases can impact the transfer ability of the native plasmids both positively and negatively (Figure 4).

We also found three other relaxase genes in the chromosome of strain NCPPB 3335, two MOB_P_ and one MOB_H_. However, they do not seem to contribute to the mobilization of the native plasmids, because derivative strains containing mutations in all three plasmidic relaxases were non-conjugative (Figure 4). This is unexpected because MOB_P_ relaxases can act in trans (Guzmán-Herrador and Llosa, 2019), and therefore it might indicate that the chromosomal relaxases preferentially act in cis or also in trans but with a very low frequency, or that they are non-functional.

The location of two relaxase genes in plasmid pC is mirrored by its possession of two functional replication initiation protein (Rep) genes (Bardaji et al., 2017a). Therefore, and because Rep genes are essential for plasmid replication, this suggest that plasmid pC originated from the cointegration of two previously independent plasmids. The presence of a truncated *traA* gene in pB, coding for the MOB_Q_ relaxase, indicate that this plasmid might have also originated from the fusion of two plasmids. The reason for the occurrence of multiple relaxase genes in these plasmids is intriguing, because plasmids with more than one replicon are common but only <2.6% of the 11386 plasmids analyzed in a previous work contain more than one relaxase gene (Coluzzi et al., 2022). It is possible then that the occurrence of these relaxases does not impart any selective advantage to strain NCPPB 3335 or its plasmids, but that it is only a neutral side effect of the fortuitous cointegration of plasmids to generate a surviving chimeric molecule. Nevertheless, we have previously shown that the presence of an additional replicon in pC contributes to a very high plasmid stability (Bardaji et al., 2019). Additionally, the data presented here support the hypothesis that the occurrence of multiple relaxase genes likely increases the chances for the horizontal transfer of virulence genes.

One possible advantage of carrying a duplicate set of determinants for replication and horizontal transfer is to maximize plasmid carriage and distribution, because of the availability of spare functional copies in the event of gene inactivation. Plasmids of *P. syringae* are subjected to repeated injury from mobile genetic elements, which could accumulate to close to a third of the plasmid sequences and can lead to gene inactivation, sequence reorganizations and internal deletions (Bardaji et al., 2011a,b). In particular, the ubiquitous insertion sequence IS*801* and diverse miniature transposable elements are the cause of frequent deletions and reorganizations of native plasmids in the *P. syringae* complex (Bardaji et al., 2011a, 2019). Additionally, incoming conjugative plasmids might differentially recognize the *oriT*-relaxase arrays of the native plasmids of strain NCPPB 3335 and facilitate their mobilization to other bacterial hosts, possibly even with a higher conjugation frequency than that achieved by the wild type strain. It is also possible that the occurrence of multiple relaxases is favored because they might impact the bacterial life cycle in other ways, instead of or in addition to their ability to transfer horizontally. In fact, relaxases can play several other functions besides their participation in conjugative transfer of their cognate *oriT* (Guzmán-Herrador and Llosa, 2019); thus, MOB_P_ and MOB_Q_ relaxases can have in trans activity on heterologous *oriT* and act as plasmid recombination enzymes. In particular, plasmid recombination may take place between related *oriT* sequences and lead to the formation or separation of cointegrates between two independent replicons (Wawrzyniak et al., 2017).

Conjugative transfer was shown to increase the stability of diverse plasmids in their native bacterial host through mobilization from plasmid-bearing to plasmid-free cells (De Gelder et al., 2007). In our conditions, however, the high rates of conjugative transfer of pB do not appear to contribute to plasmid stability. Therefore, it is likely that the main purpose of the mobilization system of strain NCPPB 3335 is the horizontal dissemination of its native plasmids. In fact, our *in vitro* experiments showed that pB can be transferred to different species of pseudomonads, all of which belong to *P. syringae sensu lato*. Additionally, pC was transferred to only two of the 20 bacteria assayed, and both belonging to the same genomospecies as strain NCPPB 3335. This suggests that the horizontal transfer of the native plasmids of strains NCPPB 3335 might be restricted or favored to closely related bacteria, as occurs with other mobile elements within the *P. syringae* complex or native plasmids from other bacteria (Smillie et al., 2010; Jackson et al., 2011; Bardaji et al., 2017b).

The acquisition of DNA by horizontal transfer has been revealed as a main factor leading to the diversification of *P. syringae* lineages (Guttman et al., 2008; Nowell et al., 2014; Dillon et al., 2019). In fact, previous works revealed incongruences between the phylogeny of diverse native plasmids and their *P. syringae* hosts (Ma et al., 2007; Bardaji et al., 2011b, 2017a; Gutiérrez-Barranquero et al., 2017), illustrating the horizontal exchange of plasmids among different pathovars. Although it is likely that this exchange was mediated by conjugation, the frequency and extent of plasmid transfer among *P. syringae* populations is unknown. Surprisingly, the conjugative transfer of native plasmids has been demonstrated in only a few strains of *P. syringae* pathovars actinidiae (Zhao et al., 2018), glycinea (Watanabe et al., 1998), papulans (Burr et al., 1988), syringae (Sundin and Bender, 1993; Cazorla et al., 2002), and tomato (Bender and Cooksey, 1986), and nearly always related to resistance to antibacterial compounds. Nevertheless, many native plasmids of *P. syringae* carry genes coding for an MPF. A hybridization analysis of 31 native plasmids of *P. syringae* showed that most of them contained genes for at least one MPF, the majority of the class MPF_T_ (previously TIVASS), although in many cases the gene clusters were incomplete (Zhao et al., 2005). The analysis of completely sequenced plasmids also supported this trend (Buell et al., 2003; Stavrinides and Guttman, 2004; Joardar et al., 2005; Bardaji et al., 2011b; Gutiérrez-Barranquero et al., 2017). These results make it likely that most native plasmids of *P. syringae* are equipped for their horizontal exchange. However, we should keep in mind that these MPFs could participate in other cellular functions instead of conjugation, such as the secretion of DNA, effectors or toxins to eukaryotic cells (Wallden et al., 2010; Souza et al., 2015). More importantly, mobilization of plasmid DNA is only possible for molecules containing an *oriT* and in the presence of a compatible relaxase, and there is no information on the abundance and distribution of origins of transfer and relaxases among *P. syringae* plasmids. To estimate the potential for horizontal gene transfer, future works should strive to evaluate the distribution of *oriT* and relaxases among native plasmids of strains of the *P. syringae* complex.

## Data availability statement

The original contributions presented in the study are included in the article/Supplementary material, further inquiries can be directed to the corresponding author.

## Author contributions

JM and CR conceived the study and designed the experiments. MA and MU performed the experiments. MA, MU, CR, and JM analyzed the data and interpreted the results. JM drafted the manuscript with contributions from MA, MU, and CR. All authors read and approved the final manuscript.
