# Supplementary material for: Multiple relaxases contribute to the horizontal transfer of the virulence plasmids from the tumorigenic bacterium Pseudomonas syringae pv. savastanoi NCPPB 3335
Source: Front Microbiol. 2022 Dec 12;13:1076710. doi: 10.3389/fmicb.2022.1076710 (PMC9791958; doi:10.3389/fmicb.2022.1076710)
Supplement: Supplementary file 1 [file Data_Sheet_1.PDF]

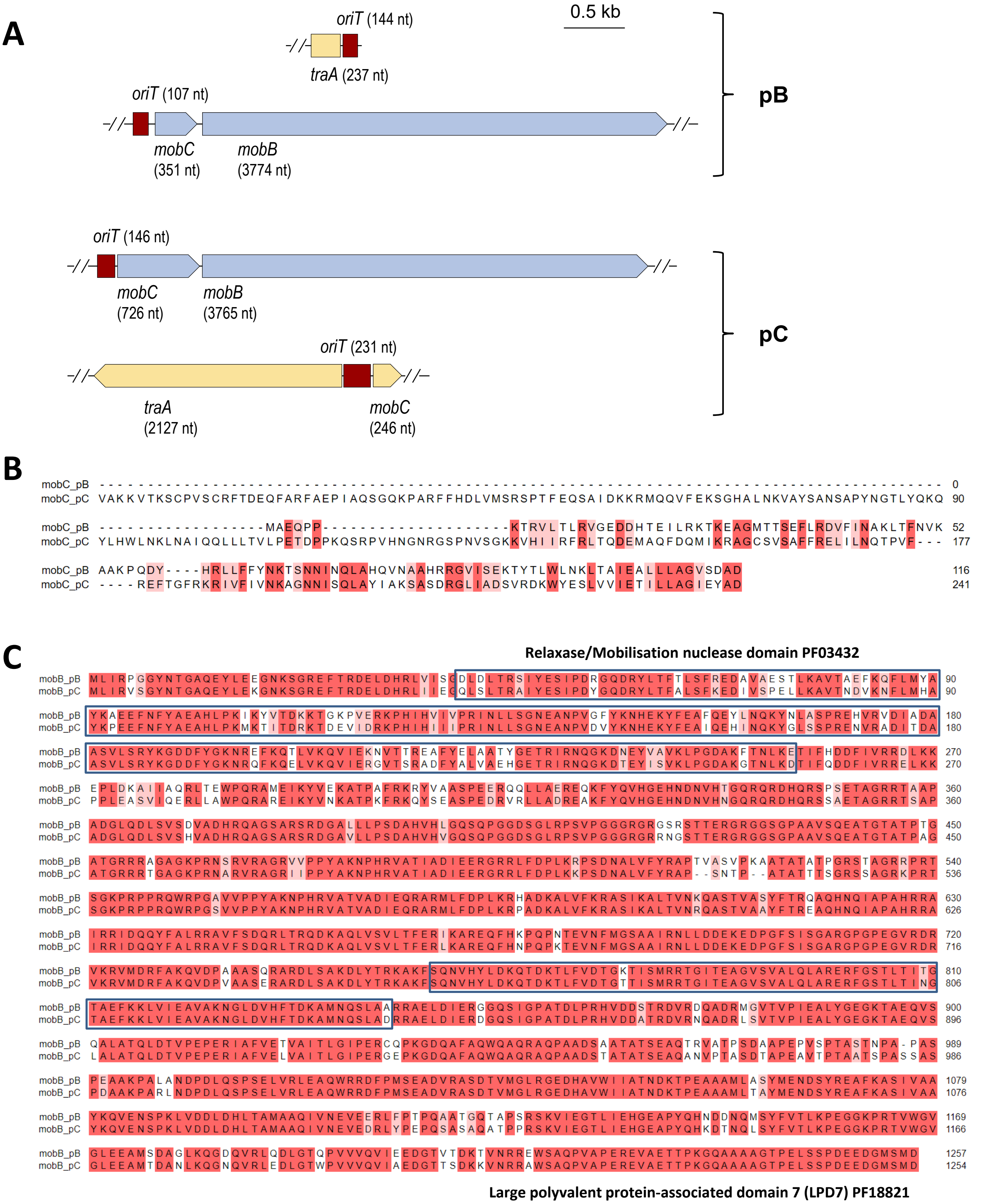


**Supplementary Figure S1.** Organization and comparison of the relaxase regions from pB and pC. **(A)** Organization of the putative origins of transfer (*oriT*) and the cognate genes for the RAP proteins (MobC) and the relaxases MobB ($MOB_P$ family) and TraA ($MOB_Q$ family). Location and extent of the *oriT* associated to *traA* are only speculative, based on origins associated to other $MOB_Q$ family relaxases. Needle alignment of the RAP proteins **(B)** and their associated $MOB_P$ relaxases **(C)** from pB and pC, as indicated to the left. Alignments were done with the Needle program at the EMBL-EBI; identical (dark red) and similar (light red) amino acids were boxed using the Sequence Manipulation Suite (http://www.bioinformatics.org/sms/). Relevant Pfam domains in the MobB proteins are boxed and described above and below the alignment in C.
