# Supplementary material for: Multiple relaxases contribute to the horizontal transfer of the virulence plasmids from the tumorigenic bacterium Pseudomonas syringae pv. savastanoi NCPPB 3335
Source: Front Microbiol. 2022 Dec 12;13:1076710. doi: 10.3389/fmicb.2022.1076710 (PMC9791958; doi:10.3389/fmicb.2022.1076710)
Supplement: Supplementary file 2 [file Data_Sheet_2.PDF]

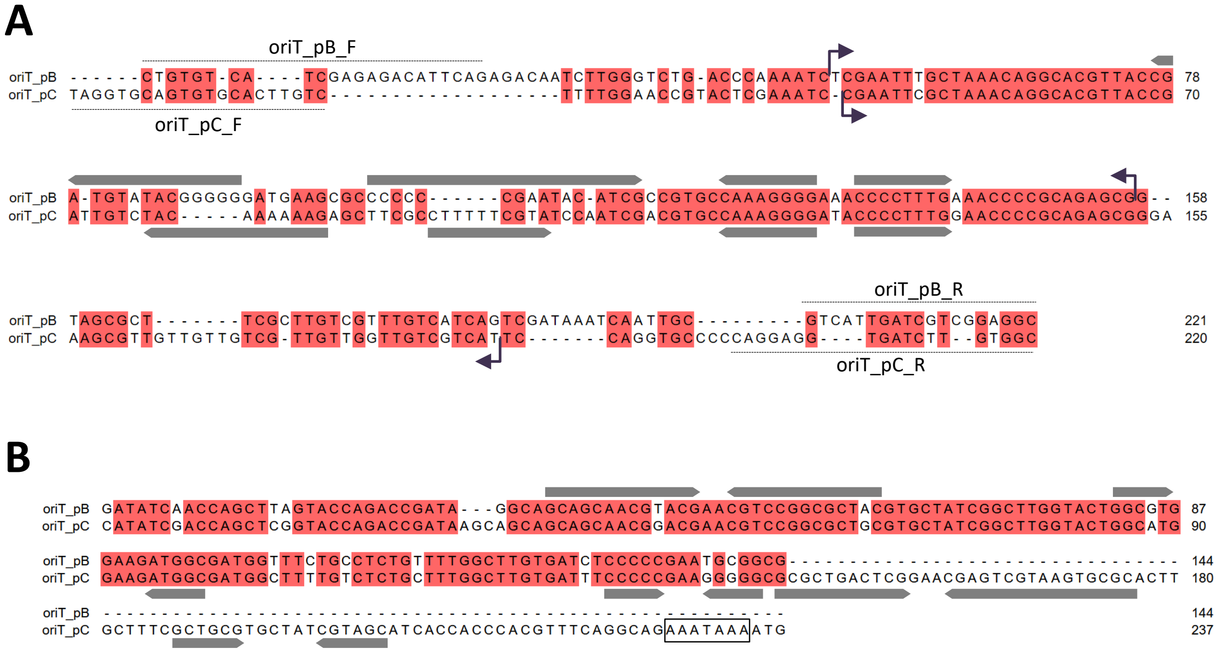

Supplementary Figure S2. Organization and functionality of the putative origins of transfer (*oriT*) from plasmids pB and pC. **(A)** Alignment of the mobilizable amplified fragments containing the *oriT* associated to the MobB relaxase genes from plasmids B (top) and C (bottom), with indication of the oligos used for amplification. The *oriT* (delimited by bent arrows) was predicted using the oriTFinder software. Identical nucleotides are highlighted in red and dashes indicate gaps introduced to maximize the alignment. **(B)** *traA-mobC* intergenic region from plasmids B and C. Inverted repeats (grey arrows) and the putative IHF-binding site (box) were predicted using the software oriTfinder, Bprom and Mfold. The sequence of *oriT*-pC starts and end with the annotated start codons of *traA* and *mobC*, respectively. Alignments were done with the Needle program at the EMBL-EBI and boxed using the Sequence Manipulation Suite (http://www.bioinformatics.org/sms/)
