# Supplementary material for: Multiple relaxases contribute to the horizontal transfer of the virulence plasmids from the tumorigenic bacterium Pseudomonas syringae pv. savastanoi NCPPB 3335
Source: Front Microbiol. 2022 Dec 12;13:1076710. doi: 10.3389/fmicb.2022.1076710 (PMC9791958; doi:10.3389/fmicb.2022.1076710)
Supplement: Supplementary file 3 [file Data_Sheet_3.PDF]

Table S1. Relevant bacterial strains and plasmids used in this study.

Abbreviated name	Collection name/Main features[a]	Reference or source
Escherichia coli		
NEB10β	Δ(*mrr-hsdRMS-mcrB*) *deoR recA1 endA1 araD139* Δ(*ara*, *leu*)7697 *galU galK* λ^- *rpsL nupG*	(Grant et al., 1990)
P. syringae **pv. savastanoi**		
WT	NCPPB 3335; pathotype strain, isolated from a diseased olive tree; contains three native plasmids (pPsv48A, pPsv48B and pPsv48C)	NCPPB[b]
Psv48ΔA	NCPPB 3335 cured of pPsv48A	(Bardaji et al., 2011)
Psv48ΔAB	NCPPB 3335 cured of pPsv48A and pPsv48B	(Bardaji et al., 2011)
FAM-098	Derivative of NCPPB 3335 containing miniTn*5*Km2 inserted in gene PSA3335_RS01855; arginine auxotroph, Km^R	(Matas et al., 2012)
ΔABC	UPN912; plasmidless derivative of strain NCPPB 3335	(Añorga et al., 2020)
ΔABCTc	UPN1265; derivative of UPN912, contains a deletion of 223 nt (positions 1216031..1216253 in accession no. NZ_CP008742) replaced by the Tc^R cassette from pHP45Ω-Tc	This work
**Plasmids**		
pA	Virulence plasmid pPsv48A of NCPPB 3335 (accession nº FR820585); 80.1 kb	(Bardaji et al., 2011)
pA::Tn*5*-GDYN1	Plasmid pPsv48A tagged with Tn*5*-GDYN1 at position 1469; Km^R, Gm^R, Sm^R, Sp^R, Suc^S	(Bardaji et al., 2011)
pB	Virulence plasmid pPsv48B of NCPPB 3335 (accession nº FR820586); 45.2 kb	(Bardaji et al., 2011)
pB(*virB4*::Km-*sacB*)	pPsv48B containing the Km^R-*sacB* cassette from pK18*mobsacB* inserted in position 19043..20180, causing a deletion of 1138 nt of the 3' end of gene *virB4*; Km^R, Suc^S	This work
pB::Tn*5*-GDYN1	pPsv48B tagged with Tn*5*-GDYN1 at position 8449; Km^R, Gm^R, Sm^R, Sp^R, Suc^S	(Bardaji et al., 2011)
pC	Virulence plasmid pPsv48C of NCPPB 3335 (accession nº FR820587); 42.1 kb	(Bardaji et al., 2011)
pB(*mobB*::Gm)	pPsv48B containing the Gm^R cassette from pJQ200SK inserted in position 38644..40842, causing a deletion of 2199 nt of gene *mobB*; Gm^R	This work

pC::Tn*5*-GDYN1	pPsv48C tagged with Tn*5*-GDYN1 inserted at position 37036; Km^R, Gm^R, Sm^R, Sp^R, Suc^S	(Bardaji et al., 2019)
pC::*sacB*	pPsv48C containing the Km^R-*sacB* cassette from pK18*mobsacB* at position 26916; Km^R, Suc^S	(Bardaji et al., 2019)
pC(*mobB*::Km)	pPsv48C containing the Km^R cassette from pK18*mobsacB* inserted in position 22259..24406, causing a deletion of 2148 nt of gene *mobB*; Km^R.	This work
pC(*traA*::Sp)	pPsv48C containing the Sp^R cassette from pHP45Ω inserted in position 38118..38574, causing a deletion of 457 nt of gene *traA*; Sp^R	This work
pDR1	Delivery vector for Tn5-GDYN1	(Flores et al., 1993)
pK18*mobsacB*	Mobilizable cloning vector, confers sucrose-dependent lethality; Km^R, Suc^S	(Schäfer et al., 1994)
pJQ200SK	Mobilizable cloning vector, confers sucrose-dependent lethality; Gm^R, Suc^S	(Quandt and Hynes, 1993)
pHP45Ω	Source of cassette Spc^R	(Prentki and Krisch, 1984)
pHP45Ω-Tc	Source of cassette Tc^R	(Fellay et al., 1987)
pKMAG	*E. coli* vector derived from pK184; 2.6 kb, Km^R; accession no. KX714576	(Bardaji et al., 2017)
pKMAGJ	pKMAG with the complete RepJ replicon from pPsv48C (coordinates 29310..31173); replicates in *E. coli* and *Pseudomonas*; Km^R	This work
pKMAGJ-*oriT*B	pKMAGJ derivative containing a 221 nt *oriT* amplicon from plasmid pB (coordinates 37837..38057); Km^R	This work
pKMAGJ-*oriT*C	pKMAGJ derivative containing a 220 nt *oriT* amplicon from plasmid pC (coordinates 21047..21266); Km^R	This work

[a] Gm, gentamicin; Km, kanamycin; Sm, streptomycin; Sp, spectinomycin; Suc^S, lethality in the presence of 5 % sucrose; Tc, tetracycline.
[b] NCPPB, National collection of Plant Pathogenic bacteria, York, United Kingdom.

References

Añorga, M., Pintado, A., Ramos, C., De Diego, N., Ugena, L., Novák, O., et al. (2020). Genes *ptz* and *idi*, coding for cytokinin biosynthesis enzymes, are essential for tumorigenesis and *in planta* growth by *P. syringae* pv. savastanoi NCPPB 3335. *Front. Plant Sci.* 11**,** 1294. doi: 10.3389/fpls.2020.01294.

Bardaji, L., Añorga, M., Echeverría, M., Ramos, C., and Murillo, J. (2019). The toxic guardians — multiple toxin-antitoxin systems provide stability, avoid deletions and maintain

virulence genes of *Pseudomonas syringae* virulence plasmids. *Mobile DNA* 10**,** 7. doi: 10.1186/s13100-019-0149-4.

Bardaji, L., Añorga, M., Ruiz-Masó, J.A., del Solar, G., and Murillo, J. (2017). Plasmid replicons from *Pseudomonas* are natural chimeras of functional, exchangeable modules. *Front. Microbiol.* 8**,** 190. doi: 10.3389/fmicb.2017.00190.

Bardaji, L., Pérez-Martínez, I., Rodríguez-Moreno, L., Rodríguez-Palenzuela, P., Sundin, G.W., Ramos, C., et al. (2011). Sequence and role in virulence of the three plasmid complement of the model tumor-inducing bacterium *Pseudomonas savastanoi* pv. savastanoi NCPPB 3335. *PLoS ONE* 6**,** e25705. doi: 10.1371/journal.pone.0025705.

Fellay, R., Frey, J., and Krisch, H. (1987). Interposon mutagenesis of soil and water bacteria: a family of DNA fragments designed for in vitro insertional mutagenesis of Gram-negative bacteria. *Gene* 52**,** 147-154. doi: 10.1016/0378-1119(87)90041-2.

Flores, M., Brom, S., Stepkowski, T., Girard, M.L., Dávila, G., Romero, D., et al. (1993). Gene amplification in *Rhizobium*: identification and *in vivo* cloning of discrete amplifiable DNA regions (amplicons) from *Rhizobium leguminosarum* biovar *phaseoli*. *Proc. Natl. Acad. Sci. USA* 90**,** 4932-4936. doi: 10.1073/pnas.90.11.4932.

Grant, S.G., Jessee, J., Bloom, F.R., and Hanahan, D. (1990). Differential plasmid rescue from transgenic mouse DNAs into *Escherichia coli* methylation-restriction mutants. *Proc. Natl. Acad. Sci. USA* 87**,** 4645-4649. doi: 10.1073/pnas.87.12.4645.

Matas, I.M., Lambertsen, L., Rodríguez-Moreno, L., and Ramos, C. (2012). Identification of novel virulence genes and metabolic pathways required for full fitness of *Pseudomonas savastanoi* pv. savastanoi in olive (*Olea europaea*) knots. *New Phytol.* 196**,** 1182-1196. doi: 10.1111/j.1469-8137.2012.04357.x.

Prentki, P., and Krisch, H.M. (1984). In vitro insertional mutagenesis with a selectable DNA fragment. *Gene* 29**,** 303-313. doi: 10.1016/0378-1119(84)90059-3.

Quandt, J., and Hynes, M.F. (1993). Versatile suicide vectors which allow direct selection for gene replacement in Gram-negative bacteria. *Gene* 127**,** 15-21. doi: 10.1016/0378-1119(93)90611-6.

Schäfer, A., Tauch, A., Jager, W., Kalinowski, J., Thierbach, G., and Pühler, A. (1994). Small mobilizable multipurpose cloning vectors derived from the *Escherichia coli* plasmids pK18 and pK19: selection of defined deletions in the chromosome of *Corynebacterium glutamicum*. *Gene* 145**,** 69-73. doi: 10.1016/0378-1119(94)90324-7.
