# Supplementary material for: Multiple relaxases contribute to the horizontal transfer of the virulence plasmids from the tumorigenic bacterium Pseudomonas syringae pv. savastanoi NCPPB 3335
Source: Front Microbiol. 2022 Dec 12;13:1076710. doi: 10.3389/fmicb.2022.1076710 (PMC9791958; doi:10.3389/fmicb.2022.1076710)
Supplement: Supplementary file 4 [file Data_Sheet_4.PDF]

**Table S2.** List and application of primers used in this work.

| Primer name | Sequence 5'-3'[a] | 5' Position | Remarks |
|---|---|---|---|
| **pPsv48B (accession no. FR820586)** | | | |
| **mobB_pB_F** | GGATCCTGCCTGTTCTTGGTTTGTTG | 37661 | Cloning *mobB* into pJET1.2 |
| **mobB_pB_R** | GGATCCTGAGCGTCACGAAGTAGCTC | 41869* | |
| **ext_mobBpB_F** | TCATCGTGGCACCCATATC | 37598 | Check marker exchange mutagenesis of *mobB* |
| **ext_mobBpB_R** | GACCTGCGTCACTCATAGCC | 41938* | |
| **virB4_F** | GGATCCTTTAACCCGCAATGGAATG | 17559 | Cloning *virB4* into pJET1.2 |
| **virB4_R** | GGATCCGGCATCAACGAAATTGAAC | 21264* | |
| **ext_virB4_F** | GAGCCTCAAGTACGGCAAAC | 17523 | Check marker exchange mutagenesis of *virB4* |
| **ext_virB4_R** | CACATCGTCCCGATGAGC | 21337* | |
| **oriT_pB_F** | TATGGTACCTGTGTCATCGAGAGACATTCAG | 37837 | Cloning *oriT* into pKMAGJ |
| **oriT_pB_R** | TATCTGCAGCCTCCGACGATCAATGAC | 38057* | |
| **bit_F** | GGATCCGCGCCGTCTGTTCTTTTTC | 15310* | Check possesion of pB and construct pB-specific probe |
| **bit_R** | GGATCCTAAGCGTGGTCGCTGATATG | 14062 | |
| **pPsv48C (accession no. FR820587)** | | | |
| **mobB_pC_F** | TACGTAAGCGTTGTTGTTGTCGTTG | 21202 | Cloning *mobB* into pJET1.2 |
| **mobB_pC_R** | TACGTATGAGCGTCACGAAGTAGCTC | 25436* | |

| | | | |
|---|---|---|---|
| **ext_mobBpC_R** | GCCTCTTCCAGCCCTACAC | 25488* | Check marker exchange mutagenesis of *mobB* with primer oriT_pC_F |
| **traA_F**<br>**traA_R** | GCGATCTGGTGCTGAGATTC<br>CATGTGGCTTTCTCCAGTTG | 37100<br>39630* | Cloning *traA* into pJET1.2 |
| **ext_traA_F**<br>**ext_traA_R** | TTCCGGACTGTTTTTGATCC<br>GCTGCTTGTTCATCGGTTTC | 37003<br>39680* | Check marker exchange mutagenesis of *traA* |
| **oriT_pC_F**<br>**oriT_pC_R** | TATGGTACCTAGGTGCAGTGTGCACTTGTC<br>TATCTGCAGCCACAAGATCACCTCCTG | 21047<br>21266* | Cloning *oriT* into pKMAGJ |
| **Other purposes** | | | |
| **Km_R**<br>**pk18mob_R** | CTAGCGAACCCCAGAGTCC<br>CAGGAAACAGCTATGACA | Used to amplify the Km$^R$ cassette from pK18*mobsacB* | |
| **pk18mob-km-sac_F**<br>**pk18mob-km-sac_R** | GATATCTTATGGACAGCAAGCGAACC<br>GATATCTCGTGGACTATGAGCACGTC | Used to amplify the Km$^R$-*sacB* cassette from pK18*mobsacB* | |
| **Gm_pJQ200_F**<br>**Gm_pJQ200_R** | GCAAAAGAAAATGCCGATG<br>CCTTGCCGTAGAAGAACAGC | Used to amplify the Gm$^R$ cassette from pJQ200SK | |
| **3335_F1** | TTTGCGCACAGCTTAGTACG | | |

| | | |
|---|---|---|
| **3335_R1** | GAATTCCTCGGGGCACAACAGGAC | Used to amplify the downstream region of pseudogene PSA3335_RS06050 from chromosome of NCPPB 3335 for insertion of $Tc^R$ cassette by marker exchange |
| **3335_F2** | GAATTCCCCGGGACATTGGCCAGTCCGAAGAC | Used to amplify the upstream region of pseudogene PSA3335_RS06050 from chromosome of NCPPB 3335 for insertion of $Tc^R$ cassette by marker exchange |
| **3335_R2** | CTTCGCACCCTTTGTTTCAG | |
| **3335_ext_F** | ATTCGTTCGGCATCGTTC | Check insertion of $Tc^R$ cassette into the pseudogene PSA3335_RS06050 from chromosome of NCPPB 3335 |
| **3335_ext_R** | CGAGGGCGACGAAAGATG | |
| **Check insertions and marker exchange** | | |
| **Km_Insrt_chk** | ACCTGCGTGCAATCCATC | |
| **Km_chk_F** | GAATGGGCTGACCGCTTC | |
| **Kmsac_chk_F** | GGTACCGAGCGAAATGACC | |
| **pJQ200SK_F** | AGGTCTGGATTTCGATCACG | |
| **Gm_pJQ200_R1** | TACGTGCAAGCAGATTACGG | |
| **Gm_pJQ200_F1** | ACGAACCGAACAGGCTTATG | |

[a] Restriction sites introduced in primers are underlined.
* The primer anneals in the reverse strand of the DNA molecule.
