# Supplementary material for: Multiple relaxases contribute to the horizontal transfer of the virulence plasmids from the tumorigenic bacterium Pseudomonas syringae pv. savastanoi NCPPB 3335
Source: Front Microbiol. 2022 Dec 12;13:1076710. doi: 10.3389/fmicb.2022.1076710 (PMC9791958; doi:10.3389/fmicb.2022.1076710)
Supplement: Supplementary file 5 [file Data_Sheet_5.pdf]

Table S3. Putative conjugation genes found in plasmids pPsv48A (NC_019265), pPsv48B (NC_019266) and pPsv48C (NC_019292) and their identification by three software programs[a].

RefSeq locus_tag	Gene name	Coordinates	Annotation	oriTfinder[b]	SecReT4[b]	MOBscan[b]	Comment
**pPsv48A**							
PSA3335_RS00255	*traR*	63618..63956	conjugal transfer protein TraR	Y	Y		5' truncated
PSA3335_RS00270	*traT*	64716..65297	conjugal transfer protein TraT	Y	Y		
PSA3335_RS00275	*traU*	65272..68328	conjugal transfer protein	Y	Y		
PSA3335_RS00290	*traW*	69005..70225	conjugal transfer protein TraW	Y	Y		
PSA3335_RS00295	*traX*	70215..70817	conjugal transfer protein TraX	Y	Y		
PSA3335_RS00300	*traY*	70844..72859	DotA/TraY family protein	Y	Y		3' frameshifted
PSA3335_RS00310	*trbA*	73776..74975	hypothetical protein	Y	Y		
PSA3335_RS00315	*trbB*	75061..76146	thioredoxin fold domain-containing protein	Y	Y		
PSA3335_RS00320	*trbC*	76133..78421	type IV secretory system conjugative DNA transfer	Y	Y		
**pPsv48B**							
PSA3335_RS00025	-	3310..>3675	MobA/MobL family protein	N	N	Y	Partial; relaxase MOB_Q
PSA3335_RS00100	*virB1*	16184..16900	lytic transglycosylase domain-containing protein	Y	Y		
PSA3335_RS00105	*virB2*	16900..17235	hypothetical protein	Y	Y		

PSA3335_RS00110	*virB3*	17248..>17649	VirB3 family type IV secretion system protein	Y	Y		
PSA3335_RS00115	*virB4*	17585..20137	conjugal transfer protein	Y	Y		
PSA3335_RS00120	*virB5*	20134..20820	P-type DNA transfer protein VirB5	Y	Y		
PSA3335_RS00130	*virB6*	21216..22148	type IV secretion system protein	Y	Y		
PSA3335_B0027[c]	*virB7*	22093..22536	Type IVA conjugal transfer protein virB7	Y	Y		
PSA3335_RS00135	*virB8*	22573..23361	type IV secretion system protein	Y	Y		
PSA3335_RS00140	*virB9*	23351..24160	P-type conjugative transfer protein VirB9	Y	Y		
PSA3335_RS00145	*virB10*	24147..25496	TrbI/VirB10 family protein	Y	Y		
PSA3335_RS00150	*virB11*	25506..26564	P-type DNA transfer ATPase VirB11	Y	Y		
PSA3335_RS00165	*virB11*	27357..27623	hypothetical protein	Y	Y		Partial
PSA3335_RS00180	*virD4*	30506..32143	type IV secretory system conjugative DNA transfer family protein	Y	Y		
PSA3335_RS00190	*traE*	32574..34739	type IA DNA topoisomerase	N	N		
PSA3335_RS00220	*mobC*	38061..38411	relaxosome component	N	N	N	RAP
PSA3335_RS00225	*mobB*	40132..42174	hypothetical protein	Y	Y	Y	Relaxase MOB$_{P}$
**pPsv48C**							
PSPSV_RS00130	*mobC*	21262..21987	CopG family transcriptional regulator	N	N	N	RAP
PSPSV_RS00135	*mobB*	21977..25741	relaxase/mobilization nuclease domain-containing protein	N	N	Y	Relaxase MOB$_{P}$

PSPSV_RS00235	*traA*	38832..36706	MobA/MobL family protein	Y	Y	Y	Relaxase MOB_Q
PSPSV_RS00240	*mobC*	39055..39300	hypothetical protein	N	N	N	RAP

[a] **oriTFinder**, https://bioinfo-mml.sjtu.edu.cn/oriTfinder/, Li et al. (2018) Nucleic Acids Res. 46(W1), W229-W234. doi: 10.1093/nar/gky352. **SecReT4**, https://bioinfo-mml.sjtu.edu.cn/SecReT4/index.php, Bi et al. (2013) Nucleic Acids Res. 41(D1), D660-D665. doi: 10.1093/nar/gks1248. **MOBscan**, https://castillo.dicom.unican.es/mobscan/, Garcillán-Barcia et al. (2020) In: de la Cruz F. (ed) *Horizontal Gene Transfer. Methods in Molecular Biology*, vol. 2075, pp. 295-308. Humana, New York, NY. doi:10.1007/978-1-4939-9877-7_21

[b] Y and N indicate that the gene was found or not found, respectively, by each of the programs.

[c] GenBank locus-tag, because it is annotated in record FR820586, but not in the corresponding Refseq record (NC_019266).
