# Supplementary material for: Multiple relaxases contribute to the horizontal transfer of the virulence plasmids from the tumorigenic bacterium Pseudomonas syringae pv. savastanoi NCPPB 3335
Source: Front Microbiol. 2022 Dec 12;13:1076710. doi: 10.3389/fmicb.2022.1076710 (PMC9791958; doi:10.3389/fmicb.2022.1076710)
Supplement: Supplementary file 6 [file Data_Sheet_6.PDF]

Table S4. Conjugative transfer of plasmids pB and pC from *P. syringae* pv. savastanoi NCPPB 3335 to different strains and species of *Pseudomonas*.

Phgr[a]	Gsp[a]	Name	Strain[b]	Acquisition of plasmid[c]	
				**pB**	**pC**
-	-	*P. marginalis*	UPN1257	-	
-	-	*P. putida*	KT2440	-	
1	8	*P. syringae* pv. actinidiae	NCPPB3871	-	
1	3	*P. syringae* pv. berberidis	CFBP1727[PT]	+	
1	3	*P. syringae* pv. delphinii	CFBP2215[PT]	+	
1	3	*P. syringae* pv. maculicola	CFBP1657[PT]	+	-
1	3	*P. syringae* pv. tomato	CFBP1920	-	
1	3	*P. syringae* pv. tomato	DCT 6D1	-	
1	3	*P. syringae* pv. tomato	DC3000 (Rif[R] derivative of CFBP2212)	-	-
1	3	*P. syringae* pv. tomato	CFBP2212[PT]	-	-
1	3	*P. syringae* pv. tomato	CFBP1321	-	-
2	1	*P. cerasi*	CFBP8306	+	
2	1	*P. syringae* pv. pisi	974B	+	-
2	1	*P. syringae* pv. syringae	UMAF 0158	-	-
2	1	*P. syringae* pv. syringae	B728a	-	
2	1	*P. syringae* pv. syringae	CFBP 762	-	-
2	1	*P. syringae* pv. lapsa	CFBP1731[PT]	+	
3	2	*P. ficuserectae*	CFBP3224[T]	-	
3	2	*P. syringae* pv. cerasicola	CFBP6110	+	
3	2	*P. syringae* pv. dendropanacis	CFBP3226[PT]	-	
3	2	*P. syringae* pv. mellea	CFBP2344[PT]	-	-
3	2	*P. syringae* pv. mellea	CFBP4475	+	-
3	2	*P. syringae* pv. myricae	CFBP2897[PT]	+	
3	2	*P. syringae* pv. nerii	CFBP5067	+	-
3	2	*P. syringae* pv. nerii	*Psn23*	+	+
3	2	*P. syringae* pv. phaseolicola	1448A	+	-
3	2	*P. syringae* pv. photiniae	CFBP2899[PT]	+	
3	2	*P. syringae* pv. savastanoi	CFBP 2074	-	
3	2	*P. syringae* pv. savastanoi	ITM317	+	
3	2	*P. syringae* pv. savastanoi	IVIA 1624-b1	+	
3	2	*P. syringae* pv. savastanoi	NCPPB 64	+	
3	2	*P. syringae* pv. savastanoi	NCPPB 1344	+	
3	2	*P. syringae* pv. tabaci	CFBP2106[PT]	-	-
3	2	*P. syringae* pv. tabaci	CFBP4855	+	+
3	2	*P. syringae* pv. tabaci	CFBP4858	-	-
3	2	*P. syringae* pv. tabaci	CFBP4861	-	-
4	4	*P. syringae* pv. atropurpurea	CFBP2340[PT]	+	
5	9	*P. cannabina*	CFBP2341[T]	-	
5	9	*P. cannabina* pv. alisalensis	CFBP1637	-	-
6	7	*P. syringae* pv. helianthi	CFBP1643	+	
6	7	*P. syringae* pv. tagetis	CECT4430[PT]	+	
7	6	*P. viridiflava*	CECT458[PT]	+	-
7	6	*P. viridiflava*	CFBP1142	-	-
7	6	*P. viridiflava*	UPN1256	-	
11		*P. cichorii*	CFBP2101[PT]	-	
uk	uk	*P. syringae* pv. syringae (or pv. tabaci)[d]	CFBP1704	-	-

[a] Distribution in phylogroups (Phgr) and genomospecies (Gsp) as described (Gardan et al., 1999; Bull and Koike, 2015; Gomila et al., 2017; Dillon et al., 2019); uk, unknown.
[b] CECT; Colección Española de Cultivos Tipo; CFBP, Collection Française des Bactéries associées aux Plantes; NCPPB, National Collection of Plant Pathogenic Bacteria; UPN, bacterial collection from the Universidad Pública de Navarra; other strains were kindly donated by F. Cazorla, M.M. López, E. López-Solanilla, and S. Tegli. PT, pathotype strain.
[c] Symbols: +, acquisiton of plasmid pB or pC by conjugation; -, no transconjugants detected. Plasmid acquisition was confirmed by PCR and examination of plasmid profiles of at least two independent transconjugants. Empty white cells indicate the experiment was not performed.
[d] It appears as pv. syringae in the CFBP and the NCPPB (NCPPB 2618) catalogues, but as pv. tabaci in the ICMP catalogue (ICMP 4410).

References

Bull, C.T., and Koike, S.T. (2015). Practical benefits of knowing the enemy: Modern molecular tools for diagnosing the etiology of bacterial diseases and understanding the taxonomy and diversity of plant-pathogenic bacteria. *Annu. Rev. Phytopathol.* 53**,** 157-180. doi: 10.1146/annurev-phyto-080614-120122.

Dillon, M.M., Thakur, S., Almeida, R.N.D., Wang, P.W., Weir, B.S., and Guttman, D.S. (2019). Recombination of ecologically and evolutionarily significant loci maintains genetic cohesion in the *Pseudomonas syringae* species complex. *Genome Biol.* 20**,** 3. doi: 10.1186/s13059-018-1606-y.

Gardan, L., Shafik, H., Belouin, S., Broch, R., Grimont, F., and Grimont, P.A.D. (1999). DNA relatedness among the pathovars of *Pseudomonas syringae* and description of *Pseudomonas tremae* sp. nov. and *Pseudomonas cannabina* sp. nov. (*ex* Sutic and Dowson 1959). *Int. J. Syst. Bacteriol.* 49**,** 469-478. doi: 10.1099/00207713-49-2-469.

Gomila, M., Busquets, A., Mulet, M., García-Valdés, E., and Lalucat, J. (2017). Clarification of taxonomic status within the *Pseudomonas syringae* species group based on a phylogenomic analysis. *Front. Microbiol.* 8**,** 2422. doi: 10.3389/fmicb.2017.02422.
